# Innovative Applications of Marine Macroalgae Polysaccharides in Tissue Engineering and Drug Delivery: A Review Study

**DOI:** 10.1002/hsr2.71365

**Published:** 2025-11-06

**Authors:** Foroozan Jalalibidgoli, Pardis Irankhahi, Helia Hajihassani, Ali Akbar Ghotbi‐Ravandi

**Affiliations:** ^1^ Department of Cell & Molecular Biology, Faculty of Life Sciences and Biotechnology Shahid Beheshti University Tehran Iran; ^2^ Department of Plant Sciences and Biotechnology, Faculty of Life Sciences and Biotechnology Shahid Beheshti University Tehran Iran

**Keywords:** bioengineering, drug delivery, marine algae, sulfated polysaccharides, tissue design, wound dressing

## Abstract

**Background and Aims:**

Marine macroalgae are abundant sources of bioactive polysaccharides—including alginate, fucoidan, ulvan, carrageenan, and agarose—widely recognized for extensive biomedical applications. These polysaccharides exhibit remarkable biological properties, such as immunostimulatory, anticancer, antioxidant, antimicrobial, and anti‐inflammatory effects, while maintaining low toxicity.

**Methods:**

A systematic literature review identified 281 studies examining sulfated polysaccharides derived from marine algae and their implementation in bioengineering fields, including tissue engineering and drug delivery applications.

**Results:**

Marine‐derived polysaccharides exhibit biocompatibility, biodegradability, and multifunctionality, making them highly suitable for developing hydrogels, nanoparticles, microspheres, and other biomaterial platforms. Their inherent structural diversity supports targeted modification for enhanced mechanical stability, drug release kinetics, and cellular interactions.

**Conclusion:**

Polysaccharides extracted from marine macroalgae represent a versatile resource for developing biomaterials in tissue engineering and drug delivery. Their multifunctional properties and adaptability position them as key materials in advancing biomedical research and addressing complex healthcare needs.

## 1 Introduction

Algae are diverse micro‐ and macroorganisms found in various ecological conditions. Marine macroalgae, or seaweed, are photosynthetic organisms living in aquatic ecosystems. Based on their photosynthetic pigments, they are classified into three groups: Chlorophyceae (green algae), Rhodophyceae (red algae), and Phaeophyceae (brown algae). By synthesizing bioactive metabolites, these organisms tolerate different habitat conditions, such as salinity, temperature changes, UV exposure, and pollutants [1]. These groups are the primary sources of valuable polysaccharides [2].

Marine algae accumulate diverse sulfated polysaccharides in their cell walls, which vary in structure based on the carbohydrate backbone, sulfate group location, and sulphation degree [3, 4, 5, 6, 7, 8].

These polysaccharides are appealing for medical product manufacturing due to their biological activity, biocompatibility, biodegradability, and potential for structural modification [9, 10].

This review examines the applications of marine macroalgae polysaccharides (alginate, fucoidan, ulvan, carrageenan, and agarose) in bioengineering, including tissue engineering and drug delivery.

## 2 Methods

**Figure 1:**
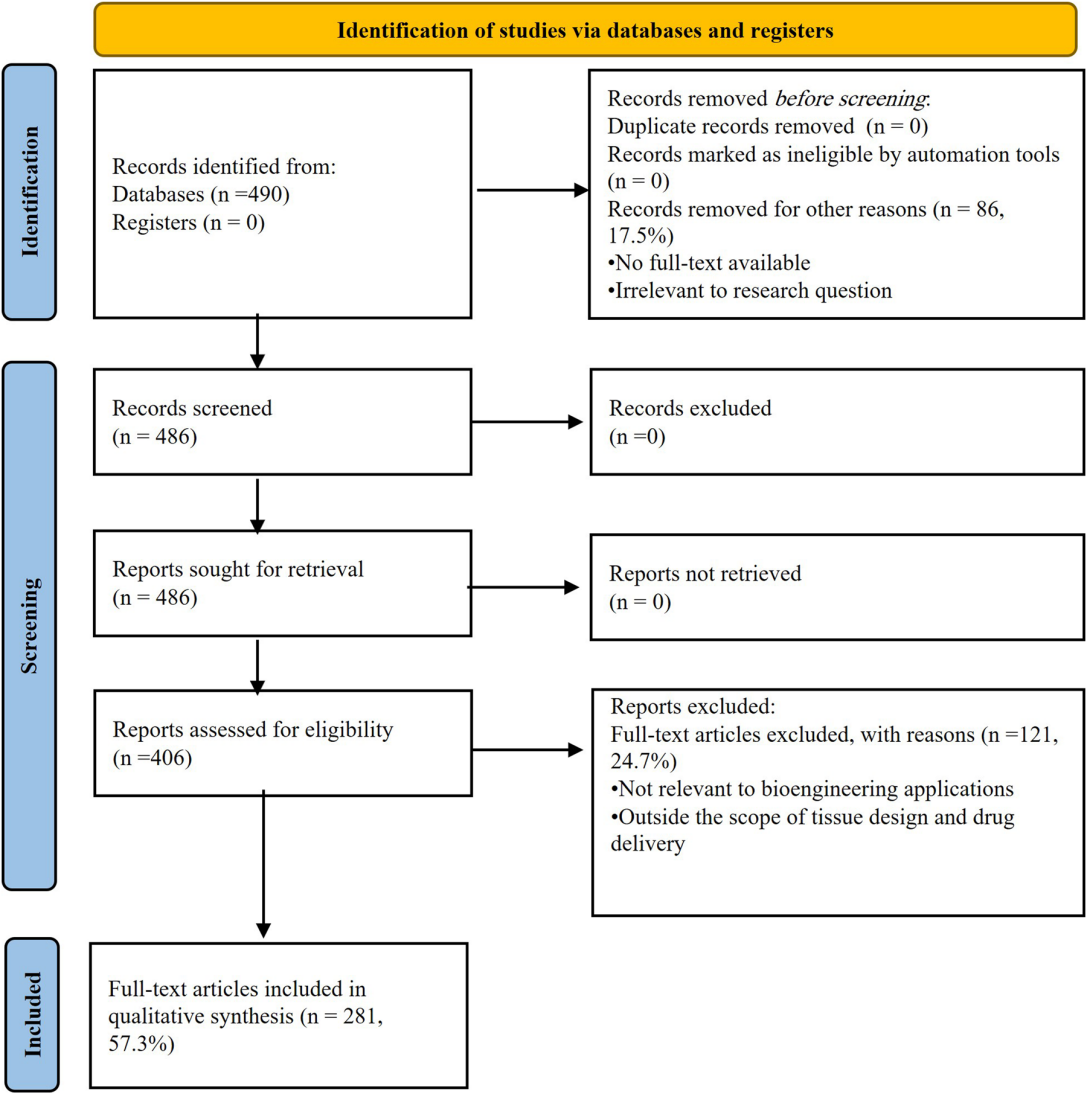
PRISMA flow diagram depicts the selection process for studies on marine macroalgae polysaccharides in bioengineering, wound healing, and drug delivery applications.

Data extraction centered on marine algal sulfated polysaccharide applications in bioengineering, focusing on properties like biocompatibility, biodegradability, and functional performance. The PRISMA protocol guided the selection, ensuring rigor and reproducibility (Figure 1). Included studies span from 2010 to 2025 and prioritize peer‐reviewed publications with clear methodology. Both in vitro and in vivo investigations showcasing biological activities, particularly immunomodulatory, antioxidant, and regenerative effects, were included.

## 3 Comparative Extraction Techniques for Marine Macroalgae Polysaccharides

Ultrasonic‐assisted extraction (UAE) enhances yield and bioactivity through cell wall disruption under mild conditions. In contrast, microwave‐assisted extraction (MAE) speeds processing but requires precise control to avoid polymer degradation. Aqueous enzymatic extraction ensures high purity by targeting cell wall components, though with longer processing times. Green extraction using organic acids such as citric acid offers an eco‐friendly alternative, reducing hazardous solvents while maintaining efficiency [11].

## 4 Marine Algae Sulfated Polysaccharides' Structure and Sources

**Figure 2:**
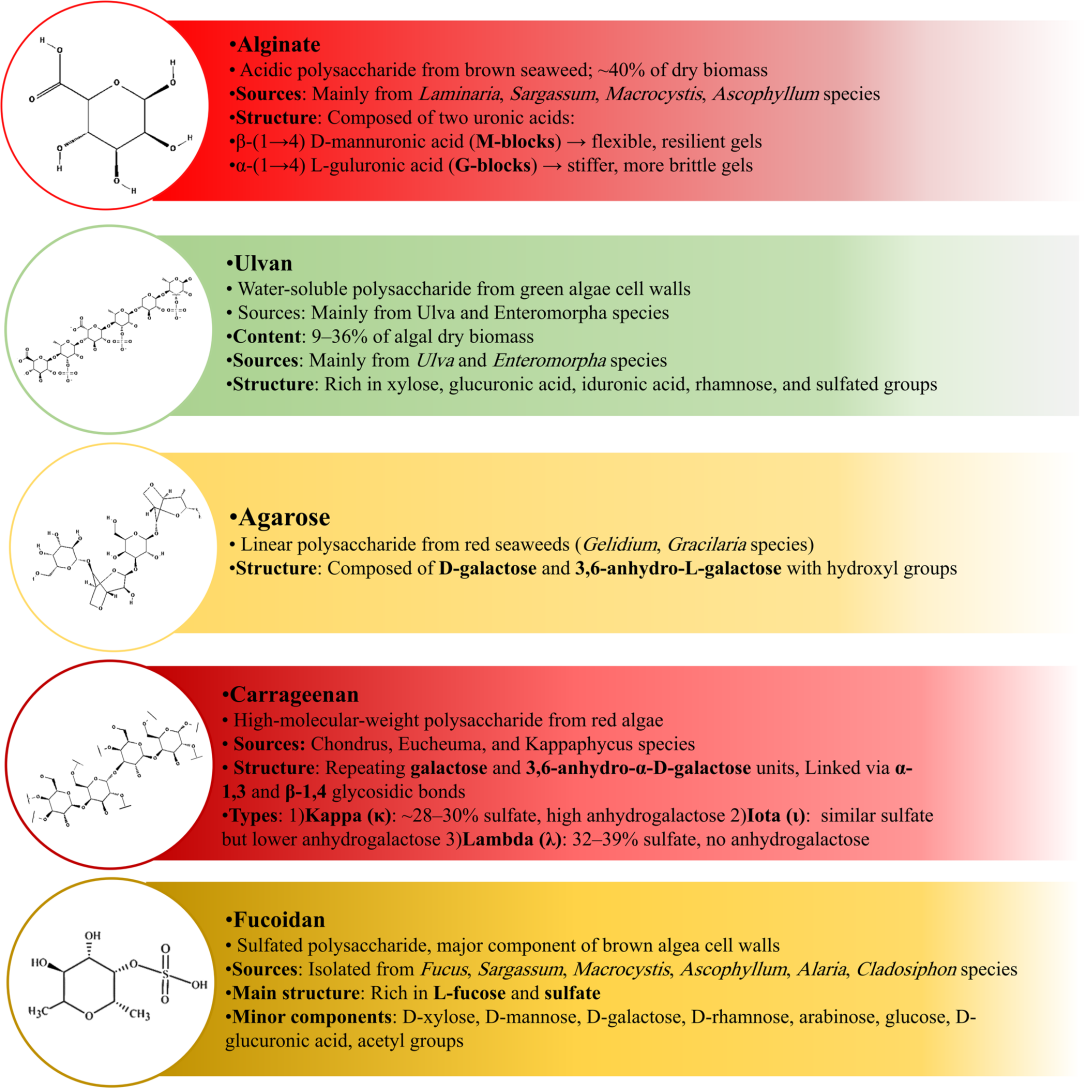
Structural characteristics, sources, and compositional details of major marine macroalgae‐derived polysaccharides: alginate, ulvan, agarose, carrageenan, and fucoidan.

Marine algae contain substantial quantities of polysaccharides within their biomass, with levels reaching 4%–76% of the organism's dry weight [12]. These marine algae sulfated polysaccharides, distinct from plant polysaccharides, possess unique physicochemical properties depending on the genus [13] (Figure 2).

Glycosaminoglycans and sulfated polysaccharides found in marine algae have also been extensively studied due to their promising properties [14]. Among the various algal polysaccharides, fucoidan, carrageenan, alginate, ulvan, and agarose are of primary interest as scaffolds, hydrogels, and wound dressings [15, 16, 17, 18].

**Figure 3:**
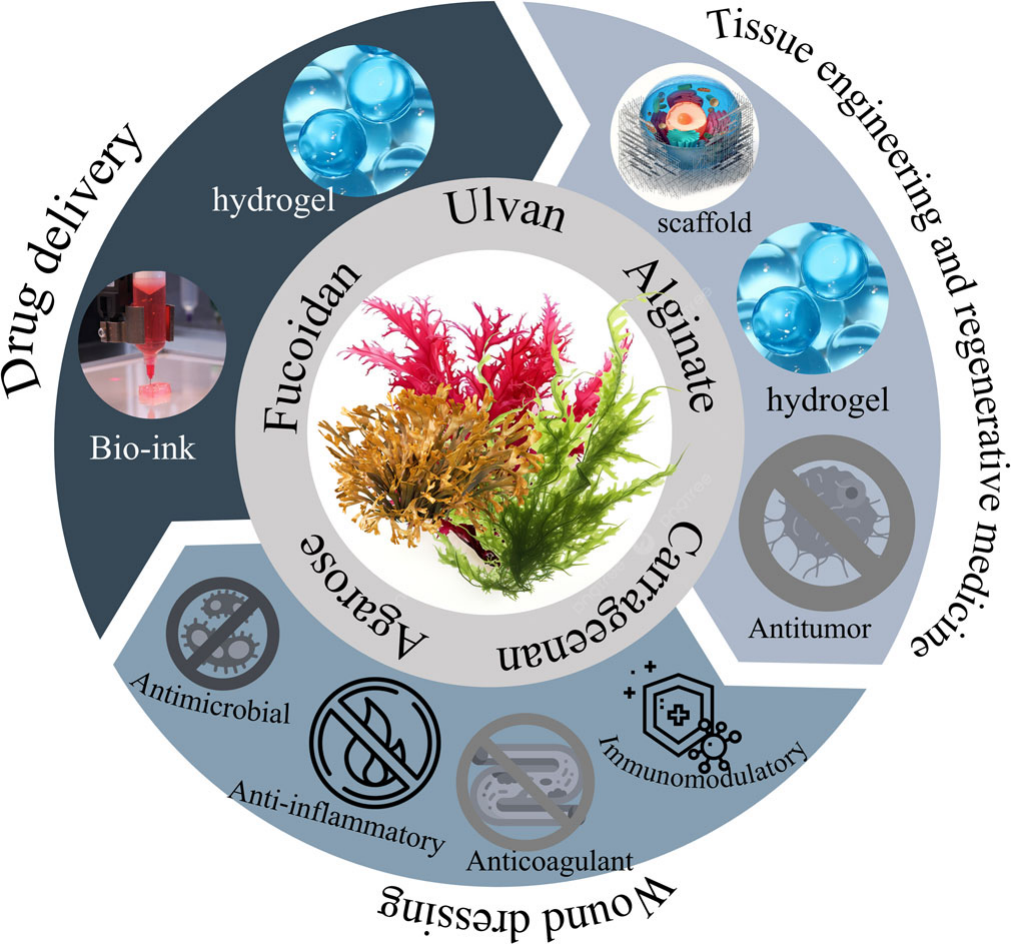
Schematic representation of marine macroalgae polysaccharides and their primary biomedical applications, including tissue engineering, regenerative medicine, wound dressing, and drug delivery.

Phaeophyceae is a rich source of fucoidan and alginates, while Rhodophyta contains carrageenan, and Chlorophyta produces anionic polysaccharides, including ulvan (Figure 3).

### 4.1 Fucoidan

Fucoidan is a sulfated polysaccharide primarily derived from brown seaweeds like *Fucus*, *Sargassum*, and *Macrocystis* [13, 19].

Fucoidan has antitumor, antivirus, anti‐inflammatory, anti‐thrombotic, and anticoagulant activities [5, 6, 20]. Fucoidan has demonstrated potential in cancer treatment, as both natural and persulfated forms inhibit VEGF165‐induced mitosis and chemotaxis in human umbilical vein endothelial cells by competitively interfering with VEGF165's interaction with the heparin‐binding domain of the KDR/Flk‐1 receptor [21].

Hepatocyte growth factor (HGF), a cytokine essential for wound healing, is produced in response to fucoidan exposure [22]. HGF promotes wound healing by increasing cell growth and movement and attracting immune cells [23].

It achieves this by downregulating the NF‐κB/MAPK pathways, reducing the levels of pro‐inflammatory cytokines such as IL‐6, TNF‐α, and IL‐1β in inflammatory models [24]. Beyond its anti‐inflammatory role, fucoidan also enhances the activity of key immune cells, including NK cells, dendritic cells, and cytotoxic T lymphocytes. Furthermore, it promotes cytokine production, such as IgG, and has shown the ability to repair immune organ damage in immunosuppressed models [25]. The immunomodulatory effects of fucoidan are closely linked to its sulfation patterns and molecular weight.

### 4.2 Alginate

Alginate is an acidic polysaccharide extracted from brown seaweeds such as *Laminaria* and *Sargassum*, making up about 40% of their dry weight [12, 26]. It is composed of β‐d‐mannuronic acid (M) and α‐l‐guluronic acid (G), which influence its gel properties—G‐rich alginates form brittle gels, while M‐rich types are more flexible [26, 27, 28].

Alginate's characteristics, such as high biocompatibility, low toxicity, low cost, non‐immunogenicity, and mild gelation by incorporating divalent ions, make it an optimal choice for wound healing, drug delivery, and regenerative medicine [29].

The scaffold is composed of sulfate/alginate. It contains TGF‐β1, PDGF‐BB, and VEGF [30]. These components have a comparable affinity for alginate‐sulfate as they do for heparin. The factor release rate of the scaffold correlates with the equilibrium binding constants, allowing VEGF, PDGF‐BB, and TGF‐β to be delivered one after the other [31]. Treating alginate‐based scaffolds with gold nanocomposites and nanowires improves electrical communication in cardiac tissue by enhancing electrical signal propagation and promoting organized tissue function [29, 32]. Alginate can form a hydrogel in the presence of metal ions such as calcium and sodium. These anionic hydrogels are applied across diverse medical contexts ([33, 34, 35, 36]; Xu, Wu et al. 2014).

### 4.3 Ulvan

Ulvan is a sulfated, water‐soluble polysaccharide from green algae such as *Ulva* and *Enteromorpha*, comprising 9%–36% of their dry weight [37, 38]. Composed mainly of xylose, glucuronic and iduronic acids, and rhamnose, ulvan exhibits anticoagulant, antifibrotic, and lipid‐lowering effects [14, 39, 40].

Antibacterial ulvan combined with marine gelatin in electrospun nanofibrous matrices accelerates wound contraction, mitigates inflammation, and aids uniform closure in burn wound models, highlighting potential in burn treatment [41, 42]. Table 1 summarizes recent progress in algal polysaccharide applications.

**Table 1:** Emerging breakthroughs and medical progress in developing wound coverage and using marine sulfated polysaccharides in wound healing.

Polysaccharide	Applications in wound healing	Administration concentration	Control setting	References
Fucidan	Fucoidan can enhance the skin healing response by increasing the expression of genes for matrix metalloproteases.	1.2% w/v (topical)	Sterile water	Anisha et al., [43]
	Fucoidan promotes the expression of key proteins involved in angiogenesis and tissue regeneration, such as VEGF, CD‐31, and α‐SMA.	1.2% w/v (topical)	Sterile water	Goushki et al., [44, 45]
	Low molecular weight Fucoidan has been shown to have anti‐inflammatory and antioxidant properties.	‐‐‐	Lipid peroxidation baseline	Li et al., [46, 47]
	Gel & Fuc‐TA hydrogel enhances vascular endothelial development factor (VEGF) expression, improves collagen synthesis, and accelerates wound repair.	0.5% w/v (EDTA)	Untreated wounds	Lu et al., [22, 48]
	Fucoidan/alginates/CMC hydrogel induces apoptosis via ROS generation.	4% polymer base + 0.5% EDTA	Saline serum control	Park et al., [22, 49, 50]
Ulvan	Incorporating Ulvan into liposomes has been investigated to improve their stability and interaction with cells, potentially enhancing their efficacy as antimicrobial delivery systems.	5% w/w (topical)	Untreated burns	Kikionis et al., [51]
	The Ulvan/PEO patch enhanced wound healing, reduced skin irritation, restored skin parameters, and decreased hemoglobin concentration and volume, with no adverse reactions.	5% w/w	Vehicle control	Ren et al., [52, 53]
	Ulvan‐AgNP hydrogel can quicken the healing of second‐degree burns and is a potential candidate for wound dressings.	1.5%–10% w/w	PIL gel base	Tziveleka et al., [54]
Alginate Agarose	SSD‐loaded alginate/chitosan constructs enhance wound healing through improved angiogenesis, antibacterial properties, and tissue regeneration. These dressings offer high exudate absorption, biocompatibility, and controlled drug delivery, making them ideal for chronic wounds, particularly in cases involving bacterial infections and diabetes‐related circulation problems.	0.75% w/v SSD	Plain hydrogel	Kim, [55, 56, 57]
	Arrangement hydrogel shows suitable properties for wound healing applications. In vitro cell development analysis also affirmed that the cells within the Alg/Chit/Hes groups, particularly those containing 10% of hesperidin, had taller cell expansion than the control group.	10% w/w hesperidin	Cell culture medium	Cruz Sánchez et al., [58, 59]
	AgNPs were generated using sodium alginate and gelatin in a hydrogel, with different concentrations of AgNO3 tested. The hydrogel demonstrated significant bactericidal action, with MICs of 0.50 µg/mL against *Pseudomonas aeruginosa* and 53.0 µg/mL against *Staphylococcus aureus*.	0.5–53 µg/mL AgNPs	Broth medium	Diniz et al., [60, 61]
	The optimal agarose concentration in water was 0.5%, balancing thickness and pH. At room temperature, both antimicrobials remained stable in the hydrogel for at least 7 days, with consistent release observed. Additionally, agarose scaffolds effectively promoted cell migration.	0.5% w/v	Culture medium	Grolman et al., [62]

### 4.4 Carrageenan

Carrageenan is a sulfated polysaccharide from red algae such as *Chondrus*, *Eucheuma*, and *Kappaphycus*, composed of alternating galactose and 3,6‐anhydro‐α‐d‐galactose units linked by α−1,3 and β−1,4 bonds [13, 63]. Its types—κappa, iota, and lambda—differ in sulfation and gel properties, making carrageenan suitable for biomedical applications due to its biocompatibility, stability, and low cost [64, 65].

Biological activities include anti‐inflammatory, antitumor, antimicrobial, antioxidant, anti‐hyperlipemia, anticoagulant, and immunomodulatory effects. It also exhibits antiviral activity against the herpes simplex virus, with activity enhanced by mild oxidation [66].

### 4.5 Agarose

Agarose is a linear polysaccharide extracted from red seaweeds such as *Gelidium* and *Gracilaria* sp. Jiang et al. [67]. Structurally, agarose consists of d‐galactose and 3,6‐anhydrous‐l‐galactose with hydroxyl groups [68].

Agarose is essential in medicine as a hydrogel for drug delivery and tissue regeneration because its antimicrobial properties hinder bacterial growth on medical device surfaces [67, 69, 70].

## 5 Biomedical Values of Algal Polysaccharides

### 5.1 Tissue‐Engineering and Wound‐Healing Applications

Bioengineering integrates engineering principles to enhance disease prevention, tissue regeneration, drug delivery systems, and energy sustainability [5, 17, 71, 72, 73, 74].

Various resins of natural or synthetic origin are used for scaffold processing [5, 33].

As a remarkable testament to the potential of seaweed‐derived polysaccharides in biomedical applications, hydrogels—3‐D hydrophilic polymer chains composed of up to 99% water—have emerged as prime examples of their widespread use in wound dressing design and tissue engineering [75]. They mimic extracellular matrix architecture and act as barriers to bacterial invasion, maintaining a favorable environment for cell viability [9]. For marine polysaccharides, functional groups such as sulfate and carboxylate enhance water absorption and support 3D structuring [32].

Hydrogels are beneficial in treating burn wounds (as wound dressing) due to their cooling properties, gas exchange, and protection against dehydration [76, 77, 78].

Alginate and chitosan offer various benefits to enhance wound care and healing. These polymers can be mixed with other polymers or ingredients to create different wound dressings, such as hydrogels, membranes, films, nanofibers, and sponges [79]. Alginate promotes faster healing by limiting scar formation and acting as a barrier to fibroblast invasion. Along with collagen, alginates are employed in tissue engineering and therapeutic settings [80].

Calcium‐alginates gel can be used in various ways, such as wound healing, tissue engineering, orthopedics, and dental implant surgery [81]. Alginate dressings release calcium ions at the wound site, activating platelets and clotting factors, thus aiding wound healing. Alginate dressings accelerate healing and improve aesthetics by promoting rapid granulation and re‐epithelialization. Moreover, they stimulate monocytes to produce excessive cytokines, including interleukin‐6 and tumor necrosis factor, which help reduce inflammation and enhance fibroblast development in mice [82].

Freeman et al. [83] and Taemeh et al. [84] proved that the implantation of sodium alginate hydrogels does not harm healthy tissue [33].

As researchers continue to explore innovative ways to harness the potential of fucoidan in biomedical applications, combining it with characteristic or engineered polymers to optimize its structure has gained considerable attention [85].

For this case, a fucoidan‐Chitosan gel was treated to quicken the healing of burn wounds in rabbits. Chitosan was utilized due to its hydrogel‐forming properties and reasons for wound dressing [7, 45].

In addition, the carrageenan‐rich ethanolic extract of the red alga *Kappaphycus alvarezii* has been shown to promote wound healing [86]. In patients with atopic dermatitis and eczema, silver‐loaded cellulosic fiber made from seaweed greatly enhanced epidermal skin physiology, barrier function, stratum corneum hydration, and surface pH over time [80].

### 5.2 Drug Delivery System

Marine polysaccharides have great potential for drug delivery systems [43, 87, 88, 89]. Sulfated polysaccharides are valuable in hydrogels, particles, and capsules because of their key properties, including nontoxicity, biodegradability, and biocompatibility, which make them suitable for various biological applications [90]. Meanwhile, their ability to form hydrogels can secure and protect the encapsulated drugs, as they dissolve in body liquids, leading to the discharge of the medication at the specified area and with the desired concentration [91].

Developing pharmaceutical nano‐formulations based on algae‐derived compounds is a promising approach for enhancing the anticancer potential of these substances, thereby offering promising therapeutic options in cancer treatment (Celso [91, 92, 93]). To address the limitations of this carotenoid in cancer treatment, various methods, like incorporating fucoxanthin into nanoemulsions, nanosuspensions, and nano‐gels, have been examined [43, 93, 94, 95, 96].

Carrageenan is the optimal polymer for twice‐daily metoprolol tartrate delivery in three‐layered matrix tablets, enhancing drug release control and linearity. Kappa carrageenan is a bio‐ink for cartilage regeneration [66, 97, 98].

Agarose hydrogels are widely used due to their biocompatibility and solute permeability in drug delivery systems, in addition to agarose pellets [99].

Marine‐derived hydrogels adapted for controlled delivery include:

- Ulvan–alginate hydrogels improved probiotic survival through the digestive tract [100, 101].
- Freeze‐dried agarose systems provided pH‐independent sustained theophylline release [102].
- Carrageenan‐based mucoadhesive films loaded with echinochrome enabled stable ophthalmic delivery [103].
- Agarose‐based pH‐responsive nanocomposites delivered quercetin with potent anticancer activity [104].
- Chitosan/pluronic/agarose hydrogels with alginate/tetracycline beads achieved sustained antibacterial release [105].

Alginate‐based systems demonstrate high potential for targeted and stimuli‐responsive delivery:

- Injectable hydrogels with > 90% loading and NIR‐triggered release suppressed tumor growth [106].
- pH‐sensitive alginate microcapsules targeted intestinal delivery [107].
- Ultrasound‐responsive alginate–Ca²⁺ hydrogels enabled on‐demand release for bone therapy [108].
- Alginate–magnetite nanoparticles enhanced curcumin solubility [109].
- ZnO–magnetic silica–alginate composites allowed pH‐responsive cancer therapy and hyperthermia‐assisted release [110, 111].
- 3D‐printed alginate scaffolds embedded with Bi₂S₃@BSA nanoradiosensitizers enhanced radiotherapy efficacy [112].

Fucoidan has been integrated into advanced delivery modalities:

- Fucoidan‐stabilized bosutinib nanocrystals improved pulmonary delivery for lung cancer [113].
- Fucoidan nanoparticles enhanced sorafenib delivery for colorectal cancer [114].
- Fucoidan–chitosan–cRGD systems enabled targeted thrombolytic therapy with reduced inflammation [115].
- ROS‐responsive fucoidan nanoparticles in dissolvable microneedles promoted macrophage repolarization in arthritis [116].
- Oxidized fucoidan hydrogels reduced inflammation, oxidative stress, and fibrosis, showing promise for adhesion prevention [117].

Table 2 provides a summary of algal sulfated polysaccharide applications in drug.

**Table 2:** Recent advances and therapeutic approaches toward using marine sulfated polysaccharides as drug delivery systems.

Polysaccharide	Applications in drug delivery	References
Fucoidan	The fucoidan hydrogels can work as bioactive compound carriers and facilitate controlled release because of their porous structure.	Anisha et al., [43]
	Fucoidan hydrogels from Turbinaria decurrens enable controlled drug release in nano‐scale shapes and exhibit antibacterial activity against both Gram‐negative and Gram‐positive pathogens.	Gao et al., [118]
	Fucoidan blended with chitosan at pH over 6 has mucoadhesive properties that enhance drug delivery to mucus membranes like the conjunctiva, nasal cavity, and vagina. This interaction with glycoproteins also supports the development of ultrasensitive diagnostic tools.	Haggag et al., [119, 120, 121]
	Fucoidan and chitosan were self‐assembled into nanocarriers targeting P‐selectin and acidic conditions in inflammatory cells. These nanocarriers enhanced drug delivery and collection in inflammatory brain injuries via intranasal administration.	Don et al., [122, 123]
	Fucoidan/chitosan and fucoidan/zein delivery systems improve solvent drug bioavailability.	Haggag et al., [120, 124]
Ulvan	A 21‐day ulvan/PEO patch treated skin trauma from cryosurgical keloid treatment, achieving significant wound healing and reduced skin inflammation. Crosslinked ulvan‐chitosan films demonstrated tensile strength of 2.23–2.48 MPa and elongation at break of 83.8%–108.5%, with suitable water vapor transmission rates for wound healing.	Don et al., [125, 126]
	Ulvan microneedles (UMNs) were improved to penetrate porcine skin to the dermis, enabling the release of model drugs like rhodamine 6 G and FITC‐BSA. With a perspective proportion of 2.63, UMNs demonstrated in situ drug release, enhanced discharge, and good biocompatibility with HaCaT and NIH3T3 cells.	Sulastri et al., [37]
	Temperature‐responsive properties have been utilized in the development of thermosensitive poly(N‐vinyl caprolactam)‐grafted aminated alginate (PNVCL‐g‐Alg‐NH2) and thermally responsive alginate‐g‐PNIPAAm copolymers. These innovations help overcome drug resistance and enhance drug delivery efficiency.	Tziveleka et al., [42]
	Alginate's ability to form structures in aqueous solutions, such as hydrogels and 3D porous scaffolds, allows for designing pharmaceutical formulations with smart targets to overcome drug side effects.	Zhong et al., [127]
Alginate	A formulation of alginate‐encapsulated liposomal bupivacaine was developed, which controlled the liposomal‐alginate concentration over time.	Aguero et al., [128]
	Alginates have been investigated for pulmonary drug delivery, and mucoadhesive microspheres have been used to prolong drug residence and expand bioavailability.	Hariyadi and Islam, [129]
	Sodium alginate microspheres were found to be suitable for ocular use, and their effectiveness improved.	Hasnain et al., [130]
	Alginate hydrogels are commonly used in drug delivery due to their ability to encapsulate drugs and release them in a controlled manner.	Park et al., [23, 58]
	These hydrogels can be tailored to provide sustained release of drugs over extended periods.	
	Alginate's mucoadhesiveness improves drug delivery with continuous release in acidic pH, making it a promising excipient. A combination with chitosan enhances mucin adhesion for tunable drug delivery carriers.	He et al., [131, 132, 133, 134]
	Agarose‐based hydrogels have been developed as pH‐sensitive carriers for drugs like cyclophosphamide, demonstrating controlled release behavior and biocompatibility in animal studies.	Shaikh et al., [127, 135]
Agarose	Agarose‐based hydrogels are porous and dissolve in alkaline environments, enhancing delivery efficiency. They can be crosslinked and controlled to manage drug diffusion, making them suitable for diverse delivery system designs.	Aslam et al., [136]
	Its temperature‐dependent properties strengthen the drug delivery system structure.	Rajabzadeh‐Khosroshahi et al., [137]
	Agarose hydrogel can serve as an on/off drug release switch in heat‐sensitive tissues. Combining MXene nanosheets with agarose creates smart hydrogels that control drug release via NIR light, adjustable by power density and exposure time.	Dong et al., [138, 139]

#### 5.2.1 Nanoformulation and Nanodelivery Developments

Marine‐derived hydrogels, including ulvan‐alginate composites, carrageenan, agarose, and fucoidan, are promising platforms for controlled drug delivery due to their biocompatibility, stimuli‐responsiveness, and tunable release kinetics [140]. Fucoidan‐based nanoparticles also provide targeted delivery capabilities in cancer and arthritis therapies, leveraging ligand‐receptor interactions for site‐specific accumulation (M [141]).

Aerogels derived from marine polysaccharides—notably chitosan, alginate, and agarose—offer ultrahigh porosity ( > 95% air volume) and nanostructured architecture ([139]; Mariangela [142]).

Hybrid systems integrating polymeric microparticles (e.g., PLGA microspheres) within hydrogel matrices enable multifunctional drug delivery [143].

Recent advances in agarose‐based hydrogels highlight how polysaccharide modification can optimize performance [144, 145, 146].

### 5.3 Therapeutic Potential of Algal Polysaccharides in Cancer, Bioengineering, and Immunology

Marine algal polysaccharides such as alginate, fucoidan, carrageenan, and agarose exhibit diverse bioactivities, including immune stimulation, anticancer effects, antioxidant properties, and antimicrobial action. Table 3 summarizes several in vitro and in vivo studies demonstrating their biomedical potential.

**Table 3:** Preclinical study cases to demonstrate the potential pharmacological activity of seaweed polysaccharides.

Class	Genus	Species	Polysaccharide	Function	Reference
Chlorophyceae	Ulva	*U. arasakii*	Ulvan	Antiaging, anticancer, and immunomodulatory activities.	Lahaye, Robic [147]
		*U. armoricana*	Ulvan	In‐vitro antibacterial activity (MIC: 0.156 − 6.25 mg/mL); immunomodulating and antiviral activity against HSV‐1 (EC50: 373.0 ± 20.7 and 320.9 ± 33.6 μg/ml); antioxidant activity (IC50: 1.8 and 12.5 mg/ml).	Berri et al., [148, 149, 150]
		*U. clathrate*	Ulvan	*In‐vitro* antiviral activity (IC_50_:0.1 μg/ml) against Newcastle disease virus (NDV)	Aguilar‐Briseño et al., [151]
		*U. compressa (*former name: *Entermorpha copmressa)*	Ulvan	*In‐vitro* antiviral activity with 100% inhibition against HSV‐1 for 100 µg/ml and 200 µg/ml.	Lopes et al., [152]
		*U. conglobate*	Ulvan	Anticoagulant, neuroprotective, and anti‐inflammatory effects	Jin et al., [153, 154]
		*U. fasciata*	Ulvan	*In‐vitro* antimicrobial activity (MIC: 8 µg/mL); antioxidant activity with 84.93% DPPH scavenging; antiarthritic activity with 86.04% maximum inhibition (IC50: 43.21%).	Barakat et al., [155]
		*U. intestinalis*	Ulvan: polysaccharide extract	*In‐vitro* antioxidant activity on DPPH (up to 73.24%) and ABTS^+^ (up to 94.94%) radicals; in‐vivo growth inhibitory activity against solid tumor formation of Sarcoma 180, with a maximum inhibitory ratio of 70.59%.	Kazemi et al., [156, 157]
		*U. lactuca*	Polysaccharide extract; Ulvan	*In‐vitro* antioxidant activity (IC_50_: 0.85) on DPPH for 100 μL of *U. lactuca* extract at 250°C; antitumoral activity on L929 cells.	Pappou et al., [158, 159]
		*U. linza Entermorpha linza*	Polysaccharide fractions	*In vitro* DPPH scavenging effect of three polysaccharide fractions showed EC50 values of 0.72, 0.65, and 0.57 mg/mL. Anticoagulant activities were influenced by sulfation degree, molecular weight, and sulfated group distribution.	Wang et al., [160]
		*U. ohnoi*	Seaweed oil	*In‐vitro* antibacterial activity against *B. subtilis, E. coli*, and *C. albicans* strains, and the DPPH radical scavenging activity in 0.7 mg/ml concentration; in‐vivo anti‐inflammatory effect.	Kang et al., [161]
		*U. pertusa*	Polysaccharide Fractions; ulvan	*In‐vitro* anticancer activity against human gastric carcinoma cell line (AGS); immunomodulatory activity; in‐vivo antioxidant activity; in‐vivo anti‐radiation activities of ulvan low molecular weight fraction.	Tabarsa et al., [162, 163, 164]
		*U. prolifera*	Ethyl acetate extract	*In‐vitro* antioxidant activity (IC_50_ = 143.18 μg/mL) in the ABTS assay.	Yao et al., [165]
		*U. rígida*	Ethanolic extract	*In‐vivo* anti‐hyperglycemic activity.	Celikler et al., [166]
Phaeophyceae	*Ascophyllum*	*A. nodosum*	Fucoidan	*In‐vitro* antioxidant activity (IC_50_: 0.35 mg/ml); reduction of inflammatory response and promotion of rapid tissue healing after a wound or surgical trauma; Anticoagulant activity.	Pereira et al., [167, 168]
	*Cladosiphon*	*C. okamuranus*	Fucoidan	*In‐vivo* immunomodulatory activity; i*n‐vitro* inhibiting proliferation and inducing cell cycle arrest in Stomach cancer.	Tomori et al., [169, 170]
	*Fucus*	*F. evanescens*	Fucoidan	*In‐vitro* and in‐vivo immunoadjuvant activity; in‐vivo inhibitory effects against endotoxin‐induced damages; i*n‐vitro* and in‐vivo anticancer effects against colorectal and lung cancers; i*n‐vitro* antiviral activity against HIV‐1 (IC_50_: 0.01 µg/ml).	Kuznetsova et al., [33, 171, 172, 173]
		*F. vesiculosus*	Fucoidan	*In‐vitro* and in‐vivo anti‐inflammatory activity; in‐vitro anti‐rotavirus in a dose‐responsive manner.	Wang et al., [174, 175]
	*Laminaria*	*L. hyperborean*	Fucoidan	*In‐vitro* coagulation‐stimulating effect of highly sulfated fucoidans in low concentrations (10 μg/mL).	Kopplin et al., [176]
		*L. japonica*	Fucoidan	Anticoagulant and antithrombotic activities; *in vitro and in vivo* antiviral activity. Fucoidan treatment of MDA‐MB‐231 and HCC1806 TNBC cell lines showed modest inhibitory activity against cell viability.	Zhao et al., [177, 178, 179, 180]
		*L. longipes*	Fucoidan	LlF fucoidan demonstrated in‐vivo anticancer activity with dosages of 100, 200, and 400 μg/mL, inhibiting colony formation by 38%, 43%, and 45% in melanoma SK‐MEL‐28 cells; 14%, 27%, and 45% in colon cancer HT‐29 cells; and 9%, 9%, and 16% in breast cancer MDA‐MB‐231 cells.	Usoltseva et al., [181]
		*L. digitate*	Alginate	A dietary supplement demonstrated that diets improved cellular and humoral immune response and cardiovascular health by raising blood lymphocytes, IgG, and IgM and lowering serum lipids.	Ribeiro et al., [182]
	*Dictyopteris*	*D. membranaceae*	Alginate	Antimicrobial activity against *Streptococcus agalactiae*, Bacillus subtilis, and Enterococcus faecium, with MICs of 17.5, 17, and 18.33 mm. In contrast, lower activity was observed against Gram‐negative bacteria and fungal strains, with MICs of 62.5‐125 μg/ml.	Akremi et al., [183]
	*Saccharina*	*S. cichorioides*	Fucoidan	The fucoidan from *S. cichorioides* and its carboxymethylated derivative can dose‐dependently decrease glucose uptake and lactate excretion in SK‐MEL‐28 melanoma cells.	Malyarenko et al., [184]
		*Saccharina latissima*	Alginate	Based on the expression of the early activation marker CD69 on the surface of CD3+ cells, the proportion of T cells activated in the presence of polysaccharide‐enriched fractions from *S. latissima* varied from 1.3% to 4.2% for all tested doses.	Moreira et al., [185]
	*Sargassum*	*S. binderi*	Fucoidan	*In‐vitro* antibacterial activity against tested bacteria in this order: *K. pneumoniae* > P. aeruginosa > *S. aureus* > S. pyogenes > B. subtilis > *E. coli*.	(S [186])
		*S. cinereum*	Fucoidan	Antiproliferative activity against HepG2, MCF‐7, and Caco‐2, and selective inhibition towards 5‐LOX over 15‐LOX.	Alzarea et al., [187]
		*S. duplicatum*	Alginate	Antioxidant activity accelerates the activities of wound‐healing processes in diabetic mice.	Ilmi et al., [188]
		*S. hemiphyllum*	Aqueous extract	*In‐vivo* hepatoprotective activity in reducing the infection rate of H. Pylori at a concentration of 250 μg/mL. Rats exposed to three different dosages (150, 300, and 600 mg/kg) of aqueous extract demonstrated considerable hepatoprotection against CCl4‐induced liver injury by lowering the initial increase of GPT and GOT levels.	Wong et al., [189]
		*S. henslowianum*	Fucoidan	*In‐vivo* immunomodulatory effect on gastric cancer rats: Promotes splenocyte proliferation and improves anti‐inflammatory cytokines (IL‐2, IL‐4, and IL‐10) secretion.	Han et al., [190]
		*S. myriocystum*	Methanolic extract	*In‐vitro* antioxidant activity with 0.645 ± 0.095 (g of ferrous sulfate equivalent/g of sample) in FRAP assay; i*n‐vitro* anti‐inflammatory screening with IC_50_ of 15.497 ± 0.16 μg/mL.	Vinodkumar, Packirisamy [191]
		*s. polycystum*	Fucoidan	*In‐vitro* antioxidant activity (65.3 ± 0.66%); i*n‐vitro* anticancer activity against MCF‐7 cell line (IC_50_:50 µg/ml)	Palanisamy et al., [192]
		*S. autumnale*	Fucoidan	*In‐vitro* anti‐inflammatory activity in a concentration‐dependent manner.	(Liyanage [193])
		*S. swartzil*	Sulfated polysaccharides extract	SPSS's potential anti‐inflammatory activity was observed to occur via the inhibition of inflammatory mediators such as NO, iNOS, COX‐2, PGE_2_, and pro‐inflammatory cytokines (TNF‐α, IL‐1β, and IL‐6).	Liyanage et al., [194]
		*S. vulgare*	Methanolic extract	*In‐vivo* immune‐stimulating and protective activities of the extract were deduced to its ability to counteract oxidative stress‐induced lipid peroxidation, modulate low levels of endogenous antioxidant enzymes like catalase (CAT) and superoxide dismutase (SOD), and suppress elevated levels of apoptotic CASP‐3 and inflammatory biomarkers like TNF‐α and IL‐6.	Ibrahim et al., [195]
		*S. natans*	Fucoidan	*In‐vitro* antioxidant activity and cytoprotective potential against Chinese fine dust (CFD)‐induced oxidative stress and apoptosis in HaCaT cells with an IC_50_ of 22.45 ± 2.73 µg mL^‐1^.	Fernando et al., [196]
		*S. aquifolium*	Alginate	*In‐vitro* antioxidant activity in DPPH(79.1%), and FRAP (86.74 μmol/100 g gallic acid) assays.	Samir et al., [197]
		*S. wightii*	Sulfated polysaccharides	The study found that Cyclosporine A, given in subcutaneous doses 5–25 mg/kg body weight over 21 days), caused hepatotoxicity in Wistar rats. The hepatotoxicity increased oxidant levels and reduced antioxidant enzyme activity. However, treatment with sulfated polysaccharides from *Sargassum wightii* restored these deformities to control levels and prevented morphological alterations.	Mancini‐Filho, Vidal‐Novoa, and Silva, [198]
Phaeophyceae	*Turbinaria*	*T. decurrens*	Ethyl acetate extract	*In‐vitro* antioxidant activity with an IC_50_ value of 180.54 μg/mL. It showed toxicity effects with LC_50_ values of 25.41 µg/mL.	Sami et al., [199]
	*Undaria*	*U. pinnatífida*	Polysaccharides extract	*In‐vitro* antioxidant activity with the greatest protective and inhibitory effects on ROS generation in H_2_O_2_‐damaged Vero cells.	Lee et al., [193]
	*Nizimuddinia*	*N. zanardini*	Aqueous extract	*In‐vitro* high levels of total antioxidant activity (70.42 ± 3.71 mg/kg), hydroxyl radical scavenging ability (81.66 ± 7.63), and ferrous ion chelating ability (76.24 ± 0.39). *Staphylococcus aureus* was the most sensitive, with an inhibitory zone diameter of 12.62 ± 0.74 mm to the aqueous extract of N. Giardini.	Moazame et al. [200]
	*Macrocystis*	*M. pyrifera*	Alginate	*In‐vivo* treatment of gastroesophageal reflux (GERD) disease; wound dressing.	Purcell et al., [201]
	*Padina*	*P. gymnospora*	Methanolic extract	*In‐vitro* wound repair, cell proliferation was blocked by 5 μg ml^−1^ Mytomycin C. Nitric oxide inhibition was quantified with Raw 264.7 by Griess reaction.	Baliano et al., [202]
		*P. antillarum*	Chloroform extract	*In‐vitro* antifungal activity against Aspergillus niger and Penicillium notatum (37 ± 0.012, 21.66 ± 0.03).	Samar et al., [203]
		*P. boryana*	Ethanolic extract	Skin whitening ability: the extract dose‐dependently inhibited the cellular melanin synthesis and tyrosinase levels.	Jayawardena et al., [204]
		*P. pavonica*	Acetonic extract	*In‐vitro* differentiation and functionality of human primary osteoblasts (Hob)	Minetti et al., [205]
		*P. tetrastromatica*	Methanolic extract	*In‐vitro* antibactericidal potential of *Padina tetrastromatica* was high against *Staphylococcus aureus t*o the extent of 96.23 ± 5.5 mm^2^, followed by *Micrococcus luteus (*87.19 ± 4.8 mm^2^) and *Enterococcus faecalis* (73.03 ± 4.7 mm^2^). The microbicidal activity produced against gram‐negative bacteria was lower, accounting for 41.63 ± 4 mm^2^, 39.63 ± 4.05 mm^2^, and 38.56 ± 4.7 mm^2^, respectively, against *Escherichia coli*, *Pseudomonas aeruginosa*, and *Klebsiella pneumoniae*.	Manilal, Mama [206]
	*Cystoseira*	*C. barbata*	70% acetone, methanol, and water extracts	The in vitro antioxidant activity and antiproliferative potential of *C. barbate's* methanol and water extracts were assessed against human tumor cell lines (pulmonary A549, colon HT‐29, mammary MCF‐7, and the non‐neoplastic breast epithelial MCF‐10A cell line). The extract containing 70% acetone from *C. barbata* (CBAE) showed the most cytotoxic and antioxidant properties.	Trifan et al., [207]
	*Colpomenia*	*C. peregrine*	Methanolic extract	This extract had in‐vitro cytotoxic activity against HCT‐116 human colon cancer cells and minimal cytotoxic effects on NCM460 non‐tumorigenic colon cells. It further inhibited the migration of HCT‐116 cells.	Al Monla et al., [208]
	*Durvillaea*	*D. antarctica*	Fucoidan	Immunomodulatory and antitumor activity.	Guerrero‐Wyss et al., [209]
	*Lessonia*	*L. nigrescens*	Seaweed powder	*In‐vivo* substantial action (60%) against Ehrlich carcinoma cancer when seaweed powder (1600 mg/kg of body weight) was administered orally for 28 days.	Deleris, Nazih, and Bard, [210]
Rhodophyceae	*Chondrus*	*C. crispus*	Methanolic extract	Neuroprotective activity, as evidenced by decreased accumulation of α‐syn and a lowered dopaminergic neuron loss in transgenic *Caenorhabditis elegans* models	Liu et al., [211]
	*Kappaphycus*	*K. alvarezii*	Carrageenan	*In‐vitro* antioxidant activity at 100 µg/mL concentration; i*n‐vitro* anticancer activity on colon carcinoma cell line (IC_50_:73.87 µg/mL)	(Chang, Okechukwu, and Teo) [212, 213]
	*Gigartina*	*G. skottsbergii*	Carrageenan	The binding capacity of carrageenan to SARS‐CoV‐2 viral particles with a yield of 8.3%.	Zank et al., [214]
	*Mastocarpus*	*M. stellatus*	Carrageenan extract (in a wide range of thermal conditions)	Rheology properties of the extract with the maximum inhibitory effect at 130°C for both the ovarian carcinoma cell line (A2780) (65%, IC50: 0.31 mg/mL) and the lung carcinoma cell line (A549) (59%, IC_50_: 0.41 mg/mL).	Flórez‐Fernández et al., [215]
	*Hypnea*	*H. musciformis*	Methanolic extract	*In‐vitro* anticancer activity: Seaweed extract treatment triggers a p53‐mediated response at the transcriptional and protein levels in liver and colon cancer cells, and the effects are more associated with metabolic changes.	Begolli et al., [216]
	*Mazzaella*	*M. laminaroides*	Carrageenan & supernatant	*In‐vitro* antiviral activity of the supernatant at concentrations between 0.156 and 5.000 g L^–1^ (EC_50_ = 0.27 g L^–1^) and the antiviral activity of carrageenan at concentrations between 0.078 and 5.000 g L^–1^ (EC_50_ = 0.09 g L^–1^).	Henríquez et al., [217]
	*Gelidium*	*Gelidium amansii*	Agar‐free extract	*In‐vivo* anti‐inflammatory and lipolysis‐promoting effects in diet‐induced obese mice.	Lee et al., [218]
	*Gracilaria*	*Gracilaria spp*.	Methanolic extract	*In‐vitro* antioxidant activity (DPPH) with an IC_50_ value of 982.25 ppm.	Hidayati et al., [219]
	*Pterocladia*	*P. capillacea*	Polysaccharides extract	*Pterocladia capillacea* showed antiviral activity against the Hepatitis C Virus in vitro and inhibitory activity against hepatocellular carcinoma HepG2 cells and peripheral blood cells infected with the virus.	Lomartire, Gonçalves [158]
	*Gelidiella*	*Gelidiella spp*.	Benzene extract	Therapeutic Potential against Alzheimer's Disease: *In‐vitro* 487.80 μg/mL of benzene extract showed significant (*p* < 0.05) inhibitory activity against both AChE and BuChE, with the percentage of inhibition 54.18 ± 5.65% (IC_50_ = 434.61 ± 26.53 μg/mL) and 78.43 ± 0% (IC_50_) = 163.01 ± 85.35 μg/mL)	Syad, Shunmugiah, and Kasi [220]
	*Ahnfeltia*	*Ahnfeltia spp*.	Ethanolic & methanolic extracts	*In‐vitro* antioxidant activity (DPPH). The ethanolic extract shows free radical scavenging activity with an IC50 value of 2 mg/mL, whereas the methanolic extract shows an IC50 value of 15.625 mg/mL.	Sah, Shankhadarwar [221]

Fucoidan, notable for its antioxidant and anticancer activities, modulates cell death pathways, inhibits tumor metastasis, and enhances chemotherapeutic efficacy [28, 222, 223]. Methacrylated κ‐carrageenan incorporated with bioactive silica nanoparticles is used in 3D bioprinted bone scaffolds exhibiting superior mechanical and osteogenic characteristics [224, 225], while agarose‐based scaffolds improved cartilage repair and regenerative medicine applications [226, 227, 228].

Alginate is widely employed in 3D‐printed scaffolds, facilitating bone and cardiac tissue regeneration, and shows promise in anticancer delivery systems [229, 230, 231]. Composite scaffolds blending fucoidan enhance mechanical strength and tissue regeneration potential [232, 233]. Targeted delivery strategies utilizing low molecular weight fucoidan demonstrate therapeutic efficacy against precancerous oral lesions [234].

Algal polysaccharides have been extensively applied in advanced wound care formulations, including hydrogels, cryogels, and composite sponges, promoting hemostasis, antibacterial activity, tissue regeneration, and modulation of macrophage phenotype in diabetic and burn wounds [235, 236, 237, 238, 239, 240]. Fucoidan‐enriched electrospun fibers and hydrogels accelerated skin wound healing and inhibited pathological angiogenesis through modulation of the Wnt/β‐catenin pathway [233, 241, 242].

Despite promising results, challenges remain regarding the precise molecular mechanisms, particularly the roles of sulfated groups and uronic acids in gut health and intracellular signaling. Standardization and regulatory hurdles also limit therapeutic translation [92, 243]. Additional in vivo studies and clinical trials are essential to validate marine polysaccharides as viable therapeutic agents and drug delivery platforms in oncology, regenerative medicine, and immunomodulation.

## 6 Prospects and Challenges

Marine algae, traditionally used in pharmaceuticals, offer promising opportunities for innovative therapies and healthcare products. Despite promises, challenges persist in understanding the structure, extraction techniques, and interactions with the human body. More research is necessary to fully explore their therapeutic mechanisms and optimize their application in complex biological systems. Despite their potential in biomedical applications, the scarcity of commercial polysaccharide products persists due to challenges with harvesting, the environment, extraction techniques, and consumer acceptance [27]. For example, although algae‐derived compounds have the potential for nanoformulations, there is limited research on their application in cancer treatment. This information scarcity highlights the need for further exploration, which may unlock new opportunities to harness these compounds' full potential in the fight against cancer [92]. Nanoparticle‐based drug delivery systems target enhanced permeability and retention (EPR), a crucial feature for cancer treatment. Using nanosized polymeric carriers, drugs can accumulate in tumor vasculature and achieve higher concentrations [43, 95]. This enhances the efficiency of anticancer compounds while lessening their side effects on nontarget cells [244]. In addition, NP carriers improve chemotherapeutic efficacy by modulating their pharmacokinetics and biodistribution profile [245, 246].

Seaweed‐derived polysaccharides, specifically alginate and fucoidan, have applications in cancer treatment and nanotechnology (for drug delivery). Therefore, they can be utilized to improve immunotherapy reactions against some diseases [247]. Their pragmatism, ease of development, and various beneficial effects against several provocative diseases and cancers make them one of the most promising and helpful choices [248].

In recent years, algae usage for purposes other than food has been quite popular. In recent years, algae usage for purposes other than food has been quite popular. Marine macroalgae polysaccharides help regulate blood glucose through several mechanisms. They inhibit carbohydrate‐hydrolyzing enzymes like α‐amylase and α‐glucosidase, slowing digestion and glucose absorption. They also enhance insulin sensitivity, improving glucose uptake in tissues. Some polysaccharides support pancreatic β‐cell function, boosting insulin secretion and glycemic control. Additionally, they influence glucose metabolism via pathways like AMPK. Understanding these effects is key to developing targeted drug delivery systems [249].

Marine macroalgae polysaccharides function as prebiotics, promoting beneficial gut microbiota while strengthening intestinal barriers and immune responses. Polysaccharide‐based nanomedicine aids microbial balance and reduces inflammation, showing promise for gut health [250]. Additionally, these polysaccharides affect the gut‐brain axis by influencing microbiota‐derived metabolites, potentially offering neuroprotective and systemic benefits in tissue engineering and drug delivery. Expanding research on these mechanisms broadens their biomedical applications ([13, 28, 143, 251, 252, 253, 254, 255, 256, 257].

Additionally, solubility constraints and chemical modification difficulties restrict the functionalization of marine polysaccharides. For instance, chitosan requires acidic environments to dissolve, and modifications may further reduce its solubility, complicating formulation [143, 258].

Moreover, while in vitro findings are promising, in vivo and clinical studies remain limited, impeding a complete understanding of pharmacokinetics, metabolism, and long‐term safety [143, 159]. The underexplored diversity of marine microbial and microalgal polysaccharides—owing to challenges in cultivation and strain identification—represents a missed opportunity to discover novel bioactive materials. Less than 1% of marine microbes are cultivable under standard conditions, and optimization of high‐yield strains remains a research priority [259].

Further, the structural complexity of marine polysaccharides demands sophisticated analytical methods for characterization, yet many studies fall short in elucidating biosynthetic pathways or structure‐function relationships [259]. Lastly, although touted as sustainable alternatives to petrochemical‐derived materials, economic and environmental challenges remain. The cost of extraction, purification, and chemical modification is often prohibitive without developing efficient, scalable, and eco‐friendly technologies [143, 145, 259, 260].

## 7 Conclusions

Marine algae are a prosperous and sustainable source of structurally diverse polysaccharides with significant biomedical potential. Compounds such as fucoidan, ulvan, alginate, carrageenan, and agarose have demonstrated various biological activities, including anti‐inflammatory, anticancer, antioxidant, and immunomodulatory effects.

However, future research must address several critical challenges to realize their therapeutic and industrial value fully. Priority areas include a more profound exploration of structure‐function relationships and mechanistic studies elucidating intracellular signaling pathways and pharmacokinetics. Expanding in vivo and clinical trials will be essential to validate efficacy and safety for human applications. Moreover, the exploration of underutilized marine microbial and microalgal sources, along with advances in cultivation and synthetic biology, may lead to the discovery of novel bioactive polysaccharides with unique functionalities.

From a translational perspective, designing next‐generation delivery systems and scaffolds that leverage marine polysaccharides' intrinsic bioactivity can accelerate their integration into clinical settings. Marine polysaccharides hold strong potential to advance regenerative medicine, immunotherapy, and targeted drug delivery.

## Author Contributions

**Foroozan Jalalibidgoli:** writing – original draft, investigation, visualization. **Pardis Irankhahi:** validation, writing – original draft, conceptualization. **Helia Hajihassani:** software, writing – original draft. **Ali Akbar Ghotbi‐Ravandi:** supervision, writing – review and editing, methodology, project administration, conceptualization.

## Ethics Statement

We affirm that this work was conceived, executed, and written in our personal and academic capacities without involvement from any government or sanctioned entity. The authors acted independently, ensuring compliance with Wiley's requirements, sanctions laws, and publishing ethics.

## Conflicts of Interest

The authors declare no conflicts of interest.

## Transparency Statement

The lead author, Foroozan Jalalibidgoli, affirms that this manuscript is an honest, accurate, and transparent account of the study being reported; that no crucial aspects have been omitted; and that any deviations from the planned approach have been fully explained.

## Data Availability

The data that support the findings of this study are available from the corresponding author upon reasonable request. Ali Akbar Ghotbi Ravandi had full access to all data in this study and takes complete responsibility for the integrity and accuracy of the data analysis.
